# Association of Eating Behaviors with Variability in Weight Change in Response to Physical Activity Interventions in Adults with Overweight

**DOI:** 10.3390/nu15153452

**Published:** 2023-08-04

**Authors:** John M. Jakicic, Renee J. Rogers

**Affiliations:** Division of Physical Activity and Weight Management, Department of Internal Medicine, University of Kansas Medical Center, Kansas City, KS 66160, USA; rrogers10@kumc.edu

**Keywords:** physical activity, overweight, weight loss, eating behavior

## Abstract

There is individual variability in weight change in response to physical activity interventions. Secondary analyses explored whether there were differences in physical activity, dietary intake, and the domains of hunger, dietary disinhibition, or dietary restraint in response to different physical activity interventions and by pattern of weight change across 6 months of an intervention. Participants (N = 207; age: 44.8 ± 8.2 years; body mass index: 27.0 ± 1.7 kg/m^2^) were included in these secondary analyses. Participants were randomly assigned to (1) a self-help physical activity intervention, (2) a prescription to progress to 150 min/week of physical activity, or (3) a prescription to progress to 300 min/week of physical activity and following 6 months were categorized based on weight change (weight gain, stability, or loss). Intervention conditions did not differ for change in weight, physical activity, dietary intake, and measures of hunger, dietary disinhibition, and total dietary restraint. Categories of weight change did not differ for change in physical activity or dietary intake, but the category of weight loss had significantly greater decreases in hunger and increases in flexible dietary restraint compared to the categories of weight stability and weight gain. The findings may provide insight into the variability in weight change in response to physical activity.

## 1. Introduction

Excess weight and adiposity are associated with chronic health conditions that include cardiovascular disease, diabetes, some forms of cancer, musculoskeletal disorders, and others [1,2]. This public health concern supports the need for interventions focused on the prevention and treatment of excess weight and adiposity. While there is a growing focus on the use of anti-obesity medications, which have been shown to be highly efficacious for weight loss [3], there remains a need to continue to focus on lifestyle factors that contribute to body weight regulation.

The 2018 United States Physical Activity Guidelines Advisory Committee reported that physical activity is associated with reduced risk for weight gain in adults, suggesting that physical activity may be important for public health efforts to prevent weight gain and the development of obesity [4]. While studies of physical activity, without also intervening on diet and energy intake, on average have resulted in modest weight loss of 0.5 kg to 3.0 kg [5,6], there appears to be variability in weight change in response to these physical activity interventions [7]. Thus, it is important to understand factors that may contribute to this variability in weight change in response to physical activity, which may inform efforts to enhance the effectiveness of physical activity to regulate body weight.

One factor that may contribute to weight regulation is whether there is a concomitant change in dietary intake and eating behaviors with physical activity [8,9]. There is some evidence that there is an acute change in energy intake in response to physical activity, and studies have also shown that there is variability in this response [10]. This variability shows that some individuals may have an increase in hunger, while others have an increase in satiety, in response to physical activity, which may contribute to the variability in weight change in response to chronic physical activity. However, studies of supervised exercise have not detected differences in energy intake in response to different volumes of physical activity that may contribute to the observed variability in weight change [11], whereas other studies have observed a difference in energy intake [12], suggesting that this warrants further investigation.

In addition to measures of energy intake being examined in studies of chronic physical activity, it may also be important to examine psychosocial constructs that may contribute to eating behavior. This may include measures of hunger, dietary disinhibition, and dietary restraint (conscious attempt to limit and monitor food intake) [13]. Studies have shown that physical activity may be positively associated with dietary restraint and negatively associated with dietary disinhibition [14,15]. However, there is a need for additional prospective studies to explore the relationship between physical activity and these domains of eating behavior and their potential contribution to the variability in weight change.

This study conducted a secondary analysis of data from an intervention study that initially examined different doses of physical activity on weight change in adults with overweight [16]. These secondary analyses were conducted to explore whether there were differences in energy intake and macronutrient composition and the domains of hunger, dietary disinhibition, or dietary restraint in response to these different physical activity interventions across the initial 6 months of treatment. Moreover, additional analyses were conducted to examine whether these outcomes and domains were associated with weight change defined as weight loss, weight stability, or weight gain following 6 months of the physical activity interventions.

## 2. Materials and Methods

### 2.1. Subjects

A subsample of the 269 adults recruited for the primary study who had baseline and 6-month data for the variables of interest were included in these secondary analyses [16]. The data for the primary study were collected between 2003 and 2009. The secondary data analyses presented in this manuscript were initiated in 2022 to explore research questions related to understanding the variability of weight change in response to physical activity.

The subsample included for these secondary analyses was 207 subjects from the primary study (77.0%) that had complete data for weight, physical activity, and the Three-Factor Eating Questionnaire at baseline and 6 months. The analysis for energy intake and macronutrient composition included 201 of these subjects (74.7% of the sample from the main study) who had complete data at baseline and 6 months for these variables. Demographic data of the 207 subjects are shown in Table 1 for each of the intervention conditions and for the overall sample. Subjects were compensated USD 50 for the completion of the 6-month outcome measures.

As previously reported, eligibility for the parent study included 18 to 55 years of age, body mass index (BMI) of 25.0 to <30 kg/m^2^, reporting < 3 days per week of <20 min per day of structured exercise, and no presence of a health condition that could confound the results or elicit safety concerns for participation [16]. All participants completed a medical history, physical activity readiness questionnaire, provided documentation from their personal physician that the proposed interventions and outcomes were not contraindicated, and provided written informed consent. The protocol and all procedures were approved by the local Institutional Review Board at the University of Pittsburgh prior to the study initiation. The secondary analyses presented here used a de-identified dataset from the parent study.

### 2.2. Outcome Measures

Measures included in the analyses presented here were collected at baseline and following 6 months of the interventions.

Weight was measured in duplicate to the nearest 0.1 kg on a calibrated scale, and height was assessed with a wall-mounted stadiometer to the nearest 0.1 cm as previously reported [16]. Weight and height were used to calculate BMI (kg/m^2^).

Physical activity was assessed with a questionnaire as previously reported [16]. The participants were queried on walking, flights of stairs climbed, and other sport, fitness, or recreational activities. Household and occupational activities were not included in this assessment. Physical activity energy expenditure was computed according to published scoring algorithms [17] with the energy expenditure for self-reported activities estimated from published data [18,19]. Minutes of physical activity were also computed.

Constructs of eating behavior were assessed using the Three-Factor Eating Questionnaire, which provides measures of dietary restraint (cognitive control of eating behavior), dietary disinhibition (disinhibition of control of eating), and hunger (susceptibility to hunger) [13]. In addition, restraint was further scored into the subscales of rigid restraint (a dichotomous all-or-nothing approach to eating, dieting, and weight status) and flexible restraint (permissive approach where foods are eaten in limited quantities without feelings of guilt) [20]. Energy intake and macronutrient composition (dietary fat, protein, carbohydrates) were assessed using a food frequency questionnaire that reflected contemporary food choices at the time the parent study was conducted [21].

### 2.3. Interventions

The parent study randomized subjects to one of three physical activity interventions as previously described [16]. This included an intervention that progressed to a prescribed dose of 150 min per week of moderate-to-vigorous intensity physical activity (MOD-PA), an intervention that progressed to a prescribed dose of 300 min per week of moderate-to-vigorous intensity physical activity (HIGH-PA), or a self-directed intervention in which a self-help manual focused on the adoption of physical activity (SELF) was provided. Both MOD-PA and HIGH-PA were offered weekly group-based intervention sessions to facilitate the adoption of their physical activity prescriptions, and during these sessions, subjects were encouraged to engage in a supervised physical activity session. All remaining weekly physical activity sessions were unsupervised. Participants in all intervention conditions were provided guidance on healthy eating behaviors and a balanced nutritional diet; however, energy restriction was not prescribed or encouraged.

### 2.4. Statistical Analysis

Statistical analyses were conducted with IBM SPSS (version 28) with statistical significance defined at *p* ≤ 0.05. Demographic characteristics are presented as means ± standard deviations for continuous variables and Ns and percentages for categorical variables.

The changes within and between intervention conditions (SELF, MOD-PA, HIGH-PA) for weight, physical activity, dietary intake, and scales of the Three-Factor Eating Inventory were analyzed using a separate one-way analysis of covariance (ANCOVA) for each variable. The ANCOVAs controlled for the baseline value of the variable being analyzed, self-identified biological sex, and self-identified race/ethnicity. Significant main effects for comparisons of intervention conditions (SELF, MOD-PA, HIGH-PA) were probed with pairwise comparisons. A similar approach was implemented for the analysis of percent weight change with the ANCOVA controlling for self-identified biological sex and self-identified race/ethnicity. The statistical significance of the change for each intervention condition was evaluated using the 95% confidence interval (95% CI), with a 95% CI not including zero deemed to be statistically significant.

Subjects were categorized based on the change in weight from baseline to 6 months. Categories include weight gain (WT-GAIN: weight gain of >3% of baseline weight), weight stability (WT-STABLE: weight change of ±3% of baseline weight), and weight loss (WT-LOSS: weight loss > 3% of baseline weight). The criterion of a 3% change in weight to define the categories is based on prior studies that have used this to define weight stability [22]. These weight change categories were compared for changes in weight, physical activity, dietary intake, and scales for the Three-Factor Eating Inventory and were analyzed using a separate one-way analysis of covariance (ANCOVA) for each variable. The ANCOVAs controlled for the baseline value of the variable being analyzed, self-identified biological sex, self-identified race/ethnicity, and intervention condition (SELF, MOD-PA, HIGH-PA). Significant main effects for comparisons of intervention conditions (WT-GAIN, WT-STABLE, WT-LOSS) were probed with pairwise comparisons. A similar approach was implemented for the analysis of percent weight change with the ANCOVA controlling for self-identified biological sex, self-identified race/ethnicity, and intervention condition (SELF, MOD-PA, HIGH-PA). The statistical significance of the change for each weight change category was evaluated using the 95% confidence interval (95% CI), with a 95% CI not including zero deemed to be statistically significant.

## 3. Results

### 3.1. Outcomes by Intervention Condition

#### 3.1.1. Weight

**Figure 1:**
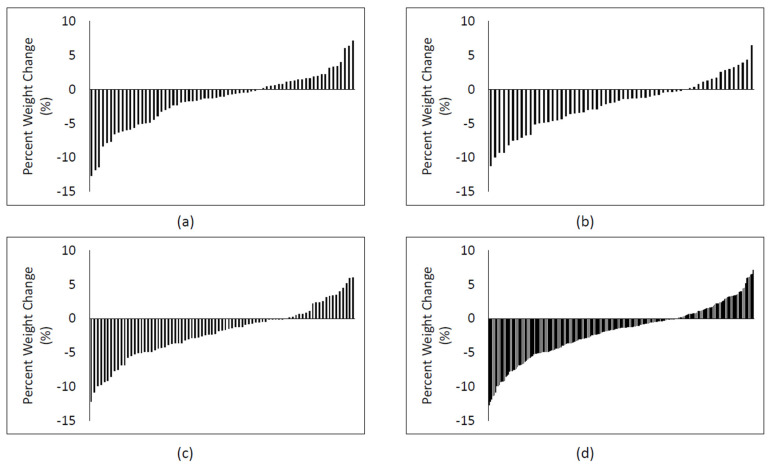
Individual 6-month percent weight change: (**a**) percent weight change for self-help physical activity intervention condition (SELF); (**b**) percent weight change for moderate-dose physical activity intervention (MOD-PA); (**c**) percent weight change for high-dose physical activity intervention (HIGH-PA); (**d**) Percent weight change for all physical activity interventions combined.

There was a modest but statistically significant decrease in weight (kg) and percent weight change in all intervention conditions (*p* < 0.05), with no significant differences between the intervention conditions (Table 2). The variability in percent weight loss by intervention conditions is shown in Figure 1. Percent weight change data were examined based on meeting the criteria for categories of WT-GAIN, WT-STABLE, and WT-LOSS (Table 3). There was no significant difference between intervention conditions for the subjects meeting each of these criteria (*p* = 0.672), with 21 (10.1%), 115 (55.6%), and 72 (34.3%) meeting the percent weight loss criteria for WT-GAIN, WT-STABLE, and WT-LOSS, respectively.

#### 3.1.2. Physical Activity

Physical activity significantly increased in SELF (715.7 kcal/week (95% CI: 471.5, 960.0)), MOD-PA (848.9 kcal/week (95% CI: 590.3, 1107.6)), and HIGH-PA (1318.3 kcal/week (95% CI: 1091.8, 1544.7)) (*p* ≤ 0.05). There was a significant difference between intervention conditions (*p* = 0.001), with post hoc analysis showing that the increase in physical activity, expressed as kcal/week, was significantly greater in HIGH-PA compared to MOD-PA and SELF (*p* ≤ 0.05). Similar results were observed when physical activity was expressed as minutes per week (Table 2).

#### 3.1.3. Dietary Intake

Dietary intake is shown in Table 3, with complete data available for 201 of the 207 subjects included in these secondary analyses. Energy intake (kcal/day) significantly decreased in all intervention conditions (*p* ≤ 0.05), with no significant difference between intervention conditions (*p* = 0.239). Analysis of the percentage of macronutrient intake showed that percent protein intake significantly increased in MOD-PA (*p* ≤ 0.05), percent carbohydrate intake significantly increased in HIGH-PA (*p* ≤ 0.05), and percent fat intake decreased in MOD-PA and HIGH-PA (*p* ≤ 0.05). The change in percent macronutrient intake did not differ between intervention conditions.

#### 3.1.4. Three-Factor Eating Inventory

Data from the analysis of the Three-Factor Eating Inventory are shown in Table 3. Hunger significantly decreased in all intervention conditions (*p* ≤ 0.05), with no significant difference between intervention conditions (*p* = 0.257). Dietary disinhibition decreased in all intervention conditions (*p* ≤ 0.05), with no significant difference between intervention conditions (*p* = 0.387). Total dietary restraint and rigid dietary restraint increased in all intervention conditions (*p* ≤ 0.05), with no significant difference between intervention conditions (*p* = 0.129 and *p* = 0.578, respectively). Flexible dietary restraint significantly increased in all intervention conditions (*p* ≤ 0.05), with there being a significant difference between intervention conditions (*p* = 0.040). Post hoc comparisons showed that the increase in flexible dietary restraint was significantly greater in MOD-PA than in HIGH-PA (*p* ≤ 0.05).

### 3.2. Outcomes by Weight Change Category

Categories for WT-GAIN, WT-STABLE, and WT-LOSS were compared for changes in weight, physical activity, dietary intake, and Three-Factor Eating Inventory. The baseline demographic characteristics of the subjects in each of these weight change categories are shown in Table 4.

#### 3.2.1. Weight Change

As expected based on the criteria for the three categories of weight change, there was a significant increase in weight in WT-GAIN (3.2 kg (95% CI: 2.7, 3.8)), a modest but statistically significant decrease in weight in WT-STABLE (−0.3 kg (95% CI: −0.6, −0.1)), and a significant decrease in weight in WT-LOSS (−4.6 kg (95%CI: −4.9, −4.3)) (*p* ≤ 0.05) (Table 5). The change in weight was significantly different between weight change categories (*p* < 0.001), with pairwise comparisons showing significant differences between WT-LOSS and both WT-STABLE and GAIN, and between WT-STABLE and WT-GAIN (*p* ≤ 0.05). Similar results were observed for the analysis of percent weight change.

#### 3.2.2. Physical Activity

Physical activity significantly (*p* ≤ 0.05) increased in all weight change categories (Weight Gain: 940.6 kcal/week (95% CI: 497.1, 1384.1); Weight Stability: 879.0 kcal/week (95% CI: 692.2, 1065.7); Weight Loss: 1167.8 kcal/week (95% CI: 929.4, 1406.2)), with no significant difference between intervention conditions (*p* = 0.172). A similar pattern of results was observed when physical activity was expressed as minutes per week (Table 5).

#### 3.2.3. Dietary Intake

Energy intake (kcal/day) significantly decreased in all weight change categories (*p* ≤ 0.05), with no significant difference between categories (*p* = 0.335) (Table 5). Analysis of the percentage of macronutrient intake showed that percent protein intake did not change from baseline in any of the categories. Percent carbohydrate intake significantly increased in WT-STABLE (*p* ≤ 0.05), and percent fat intake decreased in WT-STABLE and WT-LOSS (*p* ≤ 0.05). The change in percent macronutrient intake did not differ between the weight change categories (Table 5).

#### 3.2.4. Three-Factor Eating Inventory

The Three-Factor Eating Inventory results are shown in Table 5. Hunger significantly decreased in weight stability and weight loss (*p* ≤ 0.05). There was a significant difference between categories (*p* < 0.001), with the decrease in WT-LOSS being significantly different compared to both WT-GAIN and WT-STABLE (*p* ≤ 0.05).

Dietary disinhibition decreased in WT-STABLE and WT-LOSS (*p* ≤ 0.05). There was a significant difference between categories for change in dietary disinhibition (*p* = 0.004), with the decrease in WT-LOSS being significantly different compared to WT-STABLE (*p* ≤ 0.05).

Total dietary restraint significantly decreased in WT-STABLE and WT-LOSS (*p* ≤ 0.05), with a significant difference between weight change categories (*p* = 0.020). Pairwise comparisons showed that the change in total dietary restraint was significantly different between WT-LOSS and WT-GAIN. Rigid dietary restraint significantly increased in all categories (*p* ≤ 0.05), with no difference between categories (*p* = 0.886). Flexible dietary restraint significantly increased in WT-STABLE and WT-LOSS (*p* ≤ 0.05), with a significant difference between categories (*p* = 0.002). Pairwise comparisons showed that the change in flexible dietary restraint was significantly different in WT-LOSS compared to both WT-GAIN and WT-STABLE (*p* ≤ 0.05).

## 4. Discussion

This secondary analysis supported that on average, an intervention focused primarily on physical activity has a modest effect on change in weight in adults. Similar to the parent study [16], this secondary analysis showed that the average weight loss in the three physical activity intervention conditions was approximately 1% to 2% of baseline weight at 6 months. This magnitude of weight change is similar to that in other studies focused on physical activity or structured exercise, without a prescribed reduction in energy intake [23]. This is also consistent with literature reviews supporting that the average weight loss is typically less than 3% of baseline weight [5,6,24]. In this current study, these changes in weight did not differ by intervention condition, despite the HIGH-PA intervention resulting in a greater increase in physical activity than either MOD-PA or SELF. Moreover, this study mostly found that there were no differences between the intervention conditions for change in energy intake, macronutrient intake, or the domains of hunger, dietary disinhibition, total dietary restraint, and rigid dietary restraint. However, despite a modest but significant difference in flexible dietary restraint between MOD-PA and HIGH-PA, this did not result in a difference in weight change.

Despite the lack of differences in weight loss between the intervention conditions, this study showed variability in weight change in response to the intervention conditions, with some subjects having weight gain, weight stability, or weight loss. This difference in weight change does not appear to be a result of differences in physical activity, as this study showed that all categories of weight change had similar increases in physical activity. Thus, this study further explored whether eating behaviors may have contributed to differences in weight change and did not detect differences in energy intake between the weight change categories. However, self-report was used to assess energy intake and macronutrient composition, which may have contributed to the inability to detect meaningful and statistically significant differences. The inability to detect differences in energy intake in response to supervised exercise was also reported in studies that have used cafeteria-style measures that involved plate waste [11]. However, studies using doubly labeled water for the assessment of energy intake have reported differences between individuals who have lost more weight in response to physical activity compared to those who have lost less weight [8]. This may suggest the need for appropriate and objective measures of energy intake and macronutrients, if possible, to further explore weight change responses to physical activity.

King et al. examined compensators (less weight loss) and non-compensators (greater weight loss) in response to a 12-week supervised exercise intervention [12]. Those who were identified as compensators were found to have an increase in daily energy intake and subjective ratings of hunger compared to those who were identified as non-compensators. Results from the E-MECHANIC study also showed that individuals with less weight loss in response to exercise may have compensated with greater energy intake and increased appetite [8]. Moreover, Unick et al. showed that there was variability in response to an acute bout of exercise, with some decreasing energy intake when compared to an acute sedentary control condition [10]. The results of this current study did not detect differences in energy intake between the categories of weight change; however, there were decreases in susceptibility to hunger, which appears to be consistent with the findings in these other studies. The collective findings from these studies may provide insights pertinent to the understanding of variability in weight change in response to physical activity. However, the systematic review and meta-analysis conducted by Beaulieu et al. [9] demonstrates that most studies examining energy intake in response to physical activity and exercise are rated as fair-to-poor quality, and study quality may influence the findings pertinent to energy intake response to physical activity and exercise. This supports the need for additional high-quality studies in this area of investigation.

The systematic review and meta-analysis conducted by Beaulieu et al. [9] also reported that there is an increase in hunger and dietary restraint and a decrease in dietary disinhibition in response to exercise training. In contrast, this current study demonstrated a reduction in hunger in all the physical activity intervention conditions. However, the findings of this study were consistent with the findings of Beaulieu et al. [9] in demonstrating a reduction in dietary disinhibition and an increase in dietary restraint in all of the physical activity intervention conditions. Additional analyses of weight change categories (WT-GAIN, WT-STABLE, WT-LOSS) demonstrated that the outcomes may differ by pattern of weight change in response to physical activity. Particularly, individuals who demonstrate a reduction in weight following a 6-month physical activity intervention had a greater decrease in hunger and dietary disinhibition, and a greater increase in dietary restraint, compared to individuals who remained weight-stable or experienced weight gain. Moreover, the increase in dietary restraint associated with weight loss in response to physical activity may be influenced by a greater increase in flexible dietary restraint compared to rigid dietary restraint as demonstrated in this study. In contrast, Conlin et al. concluded that both flexible and rigid restraint can be effective for weight loss in response to resistance exercise training [25]. Westenhoefer et al. reported that flexible restraint was associated with greater weight loss and rigid restraint was associated with less weight loss in response to 6 months of a commercial weight loss program [26].

There are additional important findings from this study that warrant consideration. First, while the average weight loss across the physical activity interventions was approximately 1% to 2%, the individuals identified as meeting the criteria for weight loss (>3% decrease in weight compared to baseline weight) had an average weight loss of approximately 6%. It has been suggested that weight loss of at least 5% is associated with improvements in a variety of health-related outcomes [1]. The findings from this study suggest that a portion of adults with overweight who initiate a physical activity intervention will achieve a weight loss of at least 5%, which may improve health beyond the independent benefits of physical activity. Another potentially important finding is that the dose of physical activity observed in this study may need to be accompanied by changes in eating behavior (hunger, dietary disinhibition, dietary restraint) to result in weight loss of greater than 3% of baseline weight. By comparison, the review conducted for the 2008 Physical Activity Guidelines for Americans demonstrated that for physical activity to result in weight loss, the dose of physical activity may need to be at least 180 min per week, without being accompanied by the prescription of a reduced energy intake diet [6]. This current study extends those findings and suggests that even when a reduction in energy intake is not prescribed, physical activity may need to be accompanied by changes in domains of eating behavior. However, it is unclear if these changes in domains of eating behavior are intentionally made by the individual or whether these are an unintentional response that is influenced in another manner by physical activity, and this warrants further investigation.

The findings of this study should be considered within the context of its potential limitations. This was a secondary analysis of data with participants grouped by weight change status post hoc, and this may have allowed for unmeasured bias that influenced these findings, which warrants consideration. This study also included a self-report of physical activity that did not include measures of sedentary behavior, occupational physical activity, or household physical activity. Thus, some components of physical activity were unmeasured, which, combined with the self-reported nature of the assessment completed, may have influenced these findings. This may suggest the need to include valid and reliable measures of energy expenditure and all components of physical activity in future studies that examine the effects on weight change. This study also used a self-reported measure of energy intake, and future studies should consider other methods of assessment. However, despite their objective nature, even direct observation, plate waste measures, and the use of doubly labeled water have limitations that will also need to be considered for the assessment of energy intake. The inclusion criteria for this study also limited the sample to adults within a BMI range of 25 to <30 kg/m^2^ and within an age range of 18–55 years, and therefore, this study cannot determine if these findings would apply to individuals outside of these BMI and age ranges. Moreover, future studies should allow for the evaluation of these outcomes in response to physical activity within a sample of individuals with diversity in sex, race, and ethnicity.

## 5. Conclusions

This study reported that there is variability in weight change in response to a 6-month physical activity intervention. These findings suggest that this variability may not be fully explained by differences in physical activity participation, but rather may be accompanied by different responses in eating behavior that include hunger, dietary disinhibition, and restraint. These factors warrant consideration in future studies examining the effects of physical activity on changes in weight and body composition. Moreover, these findings may have clinical implications, and may suggest the need to assess and monitor these eating behavior outcomes in patients undertaking physical activity with the intent of weight loss. Interventions may need to include strategies that are tailored to modifying these eating behavior domains to enhance the effectiveness of physical activity for weight loss.

## Figures and Tables

**Table 1:** Summary of demographic characteristics of subjects by intervention condition.

Variable	Intervention Condition			Total
	SELF	MOD-PA	HIGH-PA	
Number of Subjects	68	60	79	207
Gender (Females)	N = 61, 89.7%	N = 55, 91.7%	N = 72, 91.1%	N = 188, 90.8%
Age (years)	44.4 ± 8.3	44.1 ± 8.4	45.7 ± 7.8	44.8 ± 8.2
Weight (kg)	74.4 ± 8.4	74.5 ± 8.3	74.3 ± 8.0	74.4 ± 8.2
Body Mass Index (kg/m^2^)	27.1 ± 1.7	27.2 ± 1.8	26.9 ± 1.6	27.0 ± 1.7
Ethnicity				
American Indian or Alaska Native	N = 1, 1.5%	N = 0, 0%	N = 0, 0%	N = 1, 0.5%
Asian	N = 1, 1.5%	N = 3, 5.0%	N = 1, 1.3%	N = 5, 2.4%
Black or African-American	N = 11, 16.2%	N = 6, 10%	N = 13, 16.5%	N = 30, 14.5%
Hispanic, Latino, Portuguese, Cape Verdean	N = 2, 2.9%	N = 1, 1.7%	N = 1, 1.3%	N = 4, 1.9%
Native Hawaiian or Other Pacific Islander	N = 0, 0%	N = 0, 0%	N = 0, 0%	N = 0, 0%
White	N = 52, 76.5%	N = 49, 81.7%	N = 63, 79.7%	N = 164, 79.2%
Other	N = 1, 1.5%	N = 1, 1.7%	N = 1, 1.3%	N = 3, 1.4%

SELF: self-help intervention; MOD-PA: moderate-dose physical activity intervention; HIGH-PA: high-dose physical activity intervention.

**Table 2:** Change in weight, physical activity, dietary intake, hunger, dietary disinhibition, and dietary restraint by intervention condition.

Variables	Baseline (Mean (95% Confidence Interval))	Change from Baseline to 6 Months (LS Mean (95% Confidence Interval)) *	*p*-Values **
Body Weight (kg)			0.549
SELF (N = 68)	74.2 (72.4, 76.0)	−1.1 (−1.8, −0.4)	
MOD-PA (N = 60)	74.6 (72.7, 76.5)	−1.6 (−2.3, −0.9)	
HIGH-PA (N = 79)	74.3 (72.7, 76.0)	−1.6 (−2.2, −0.9)	
Percent Weight Change (%)			0.600
SELF (N = 68)	-----	−1.5 (−2.5, −0.6)	
MOD-PA (N = 60)	-----	−2.1 (−3.1, −1.1)	
HIGH-PA (N = 79)	-----	−2.1 (−3.0, −1.2)	
Physical Activity (kcal/week)			0.001
SELF (N = 68)	985.1 (789.9, 1181.4)	715.7 (471.5, 960.0) ^A^	
MOD-PA (N = 60)	872.3 (663.4, 1081.1)	848.9 (590.3, 1107.6) ^B^	
HIGH-PA (N = 79)	717.4 (535.5, 899.3)	1318.3 (1091.8, 1544.7) ^A,B^	
Physical Activity (min/week)			0.002
SELF (N = 68)	140.0 (103.9, 176.1)	132.8 (88.5, 177.1) ^A^	
MOD-PA (N = 60)	121.5 (83.2, 159.9)	161.3 (114.4, 208.3) ^B^	
HIGH-PA (N = 79)	87.5 (54.0, 120.9)	240.0 (198.9, 281.2) ^A,B^	
Energy Intake (kcal/day)			0.239
SELF (M = 66)	1851.5 (1653.9, 2049.0)	−173.8 (−296.2, −51.3)	
MOD-PA (N = 58)	1809.2 (1597.1, 2021.2)	−328.1 (−458.7, −197.5)	
HIGH-PA (N = 77)	1823.0 (1639.9, 2006.1)	−248.6 (−361.8, −135.3)	
Percent Protein Intake (%)			0.152
SELF (M = 66)	15.7 (15.1, 16.4)	0.3 (−0.3, 0.9)	
MOD-PA (N = 58)	15.4 (14.6, 16.1)	1.0 (0.4, 1.6)	
HIGH-PA (N = 77)	15.0 (14.4, 15.7)	0.3 (−0.3, 0.8)	
Percent Carbohydrate Intake (%)			0.199
SELF (M = 66)	46.4 (44.3, 48.5)	0.8 (−1.0, 2.6)	
MOD-PA (N = 58)	48.8 (46.5, 51.1)	1.5 (−0.4, 3.4)	
HIGH-PA (N = 77)	45.2 (43.3, 47.2)	2.9 (1.3, 4.6)	
Percent Fat Intake (%)			0.094
SELF (M = 66)	38.3 (34.4, 40.3)	−0.8 (−2.3, 0.7)	
MOD-PA (N = 58)	36.5 (34.5, 38.6)	−2.0 (−3.6, −0.4)	
HIGH-PA (N = 77)	40.1 (38.3, 41.9)	−3.1 (−4.5, −1.7)	
Hunger			0.257
SELF (N = 68)	6.2 (5.3, 7.1)	−1.0 (−1.6, −0.4)	
MOD-PA (N = 60)	6.4 (5.5, 7.4)	−1.6 (−2.3, −1.0)	
HIGH-PA (N = 79)	6.2 (5.4, 7.1)	−1.0 (−1.6, −0.5)	
Dietary Disinhibition			0.387
SELF (N = 68)	8.9 (8.0, 9.7)	−1.0 (−1.6, −0.4)	
MOD-PA (N = 60)	8.6 (7.7, 9.5)	−1.2 (−1.9, −0.6)	
HIGH-PA (N = 79)	9.1 (8.3, 9.8)	−1.5 (−2.1, −1.0)	
Dietary Restraint (Total)			0.129
SELF (N = 68)	10.1 (9.1, 11.0)	2.1 (1.3, 2.9)	
MOD-PA (N = 60)	9.5 (8.5, 10.5)	3.1 (2.3, 4.0)	
HIGH-PA (N = 79)	9.6 (8.7, 10.5)	2.1 (1.3. 2.8)	
Dietary Restraint (Rigid)			0.578
SELF (N = 68)	3.0 (2.6, 3.4)	0.8 (0.4, 1.1)	
MOD-PA (N = 60)	3.0 (2.5, 3.4)	1.0 (0.6, 1.3)	
HIGH-PA (N = 79)	2.8 (2.4, 3.2)	0.9 (0.6, 1.2)	
Dietary Restraint (Flexible)			0.040
SELF (N = 68)	3.1 (2.8, 3.5)	0.7 (0.4, 1.1)	
MOD-PA (N = 60)	2.9 (2.5, 3.3)	1.3 (0.9, 1.7) ^A^	
HIGH-PA (N = 79)	3.1 (2.8, 3.5)	0.6 (0.3, 1.0) ^A^	

SELF: self-help intervention; MOD-PA: moderate-dose physical activity intervention; HIGH-PA: high-dose physical activity intervention. * Change scores with a 95% confidence interval that does not include 0 are statistically significant at *p* ≤ 0.05. Change scores control for baseline value, intervention condition, sex, race/ethnicity (percent weight change did not control for baseline value). ** *p*-value for comparison of the change from baseline to 6 months between weight change categories controlling for baseline value, intervention condition, sex, race/ethnicity (percent weight change did not control for baseline value). The change scores for intervention conditions with the same superscript are significantly different at *p* ≤ 0.05.

**Table 3:** Distribution of weight change by intervention condition (N = 217).

Intervention Condition	Weight Change Category		
	>3% Weight Gain	Within 3% of Baseline Weight	>3% Weight Loss
SELF	7 (10.2%)	42 (61.8%)	19 (27.9%)
MOD-PA	5 (8.3%)	33 (55.0%)	22 (36.7%)
HIGH-PA	9 (11.4%)	40 (50.6%)	30 (38.0%)
Total	21 (10.1%)	115 (55.6%)	71 (32.3%)

SELF: self-help intervention; MOD-PA: moderate-dose physical activity intervention; HIGH-PA: high-dose physical activity intervention. *p*-value for Pearson chi-square = 0.672.

**Table 4:** Summary of demographic characteristics of subjects by weight change category.

Variable	Weight Change Category			Total
	WT-GAIN	WT-STABLE	WT-LOSS	
Number of Subjects	21	115	71	207
Gender (Females)	N = 61, 89.7%	N = 55, 91.7%	N = 72, 91.1%	N = 188, 90.8%
Age (years)	44.4 ± 8.3	44.1 ± 8.4	45.7 ± 7.8	44.8 ± 8.2
Weight (kg)	74.4 ± 8.4	74.5 ± 8.3	74.3 ± 8.0	74.4 ± 8.2
Body Mass Index (kg/m^2^)	27.1 ± 1.7	27.2 ± 1.8	26.9 ± 1.6	27.0 ± 1.7
Ethnicity				
American Indian or Alaska Native	N = 1, 4.8%	N = 0, 0%	N = 0, 0%	N = 1, 0.5%
Asian	N = 1, 4.8%	N = 4, 3.5%	N = 0, 0%	N = 5, 2.4%
Black or African-American	N = 7, 33.3%	N = 14, 12.2%	N = 9, 12.7%	N = 30, 14.5%
Hispanic, Latino, Portuguese, Cape Verdean	N = 0, 0%	N = 1, 0.9%	N = 3, 4.2%	N = 4, 1.9%
Native Hawaiian or Other Pacific Islander	N = 0, 0%	N = 0, 0%	N = 0, 0%	N = 0, 0%
White	N = 12, 57.1%	N = 94, 81.7%	N = 58, 81.7%	N = 164, 79.2%
Other	N = 0, 0%	N = 2, 1.7%	N = 1, 1.4%	N = 3, 1.4%

WT-GAIN: >3% weight gain from baseline weight; WT-STABLE: ±3% weight change from baseline weight; WT-LOSS: >3% weight loss from baseline weight.

**Table 5:** Change in weight, physical activity, dietary intake, hunger, dietary disinhibition, and dietary restraint by category of weight loss.

Variables	Baseline (Mean (95% Confidence Interval))	Change from Baseline to 6 Months (LS Mean (95% Confidence Interval)) *	*p*-Values **
Body Weight (kg)			<0.001
WT-GAIN (N = 21)	73.7 (70.4, 76.9)	3.2 (2.7, 3.8) ^A,B^	
WT-STABLE (N = 115)	74.4 (73.0, 75.8)	−0.3 (−0.6, −0.1) ^A,C^	
WT-LOSS (N = 71)	74.5 (72.8, 76.3)	−4.6 (−4.9, −4.3) ^B,C^	
Percent Weight Change (%)			<0.001
WT-GAIN (N = 21)	-----	4.5 (3.6, 5.3) ^A,B^	
WT-STABLE (N = 115)	-----	−0.4 (−0.8, −0.1) ^A,C^	
WT-LOSS (N = 71)	-----	−6.2 (−6.7, −5.8) ^B,C^	
Physical Activity (kcal/week)			0.172
WT-GAIN (N = 21)	874.5 (513.5, 1235.5)	940.6 (497.1, 1384.1)	
WT-STABLE (N = 115)	871.7 (719.8, 1023.7)	879.0 (692.2, 1065.7)	
WT-LOSS (N = 71)	808.2 (614.2, 1002.2)	1167.8 (929.4, 1406.2)	
Physical Activity (min/week)			0.155
WT-GAIN (N = 21)	130.6 (64.3, 196.9)	169.8 (89.3, 250.3)	
WT-STABLE (N = 115)	117.5 (89.6, 145.4)	162.9 (129.0, 196.8)	
WT-LOSS (N = 71)	105.2 (69.6, 140.8)	216.5 (173.3, 259.8)	
Energy Intake (kcal/day)			0.335
WT-GAIN (N = 20)	2061.5 (1700.5, 2422.5)	−347.4 (−573.9, −120.9)	
WT-STABLE (N = 112)	1847.8 (1695.8, 1999.8)	−201.6 (−295.8, −107.4)	
WT-LOSS (N = 69)	1726.7 (1531.5, 1921.9)	−291.5 (−412.0, −171.0)	
Percent Protein Intake (%)			0.849
WT-GAIN (N = 20)	15.1 (13.8, 16.3)	0.3 (−0.9, 1.4)	
WT-STABLE (N = 112)	15.3 (14.8, 15.9)	0.5 (0.0, 0.9)	
WT-LOSS (N = 69)	15.5 (14.8, 16.1)	0.6 (0.0, 1.3)	
Percent Carbohydrate Intake (%)			0.215
WT-GAIN (N = 20)	48.3 (44.4, 52.2)	−0.7 (−4.2, 2.9)	
WT-STABLE (N = 112)	46.7 (45.1, 48.4)	2.6 (1.0, 4.0)	
WT-LOSS (N = 69)	46.0 (43.9, 48.1)	1.4 (−0.5, 3.2)	
Percent Fat Intake (%)			0.222
WT-GAIN (N = 20)	37.6 (34.0, 41.2)	0.1 (−2.9, 3.2)	
WT-STABLE (N = 112)	38.7 (37.2, 40.2)	−2.6 (−3.9, −1.3)	
WT-LOSS (N = 69)	38.4 (36.5, 43.4)	−1.6 (−3.3, −0.1)	
Hunger			<0.001
WT-GAIN (N = 21)	6.6 (5.0, 8.2)	0.4 (−0.7, 1.4) ^A^	
WT-STABLE (N = 115)	6.4 (5.7, 7.1)	−1.0 (−1.4, −0.6) ^B^	
WT-LOSS (N = 71)	6.0 (5.1, 6.9)	−2.0 (−2.5, −1.4) ^A,B^	
Dietary Disinhibition			0.004
WT-GAIN (N = 21)	9.4 (7.9, 10.9)	−0.4 (−1.5, 0.6)	
WT-STABLE (N = 115)	9.0 (8.3, 9.6)	−0.9 (−1.4, −0.5) ^A^	
WT-LOSS (N = 71)	8.5 (7.7, 9.3)	−2.0 (−2.6, −1.5) ^A^	
Dietary Restraint (Total)			0.020
WT-GAIN (N = 21)	9.5 (7.7, 11.3)	0.8 (−0.7, 2.3) ^A^	
WT-STABLE (N = 115)	9.7 (8.9, 10.4)	2.2 (1.6, 2.8)	
WT-LOSS (N = 71)	9.9 (8.9, 10.8)	3.1 (2.3, 3.9) ^A^	
Dietary Restraint (Rigid)			0.886
WT-GAIN (N = 21)	2.6 (1.8, 3.3)	0.9 (0.3, 1.5)	
WT-STABLE (N = 115)	3.0 (2.6, 3.3)	0.8 (0.6, 1.1)	
WT-LOSS (N = 71)	2.9 (2.5, 3.3)	0.9 (0.6, 1.3)	
Dietary Restraint (Flexible)			0.002
WT-GAIN (N = 21)	3.0 (2.3, 3.7)	0.0 (−0.7, 0.7) ^A^	
WT-STABLE (N = 115)	3.1 (2.8, 3.4)	0.7 (0.5, 1.0) ^B^	
WT-LOSS (N = 71)	3.1 (2.7, 3.5)	1.3 (0.9, 1.6) ^A,B^	

Weight Gain: >3% weight gain from baseline weight; Weight Stability: ±3% weight change from baseline weight; Weight Loss: >3% weight loss from baseline weight. * Change scores with a 95% confidence interval that does not include 0 are statistically significant at *p* ≤ 0.05. Change scores control for baseline value, intervention condition, sex, race/ethnicity (percent weight change did not control for baseline value). ** *p*-value for comparison of the change from baseline to 6 months between weight change categories controlling for baseline value, intervention condition, sex, race/ethnicity (percent weight change did not control for baseline value). The change scores for weight categories with the same superscript are significantly different at *p* ≤ 0.05.

## Data Availability

Data from a de-identified dataset were used for analysis in this study. For potential sharing of this dataset, please contact the corresponding author.
